# PET Criteria by Cancer Type from Imaging Interpretation to Treatment Response Assessment: Beyond FDG PET Score

**DOI:** 10.3390/life13030611

**Published:** 2023-02-22

**Authors:** Francesco Dondi, Achille Lazzarato, Joana Gorica, Priscilla Guglielmo, Francesca Borgia, Rossella Filice, Antonio Vento, Sara Pacella, Riccardo Camedda, Matteo Caracciolo, Maria Silvia De Feo, Paolo Mammucci, Viviana Frantellizzi, Naima Ortolan, Elisa Fiasconaro, Luca Urso, Laura Evangelista, Riccardo Laudicella, Giulia Santo

**Affiliations:** 1 Nuclear Medicine, ASST Spedali Civili di Brescia, 25123 Brescia, Italy; 2 Unit of Nuclear Medicine, Department of Medicine, Surgery and Pharmacy, Università degli Studi di Sassari, 07100 Sassari, Italy; 3 Department of Radiological Sciences, Oncology and Anatomo-Pathology, Sapienza, University of Rome, 00161 Rome, Italy; 4 Nuclear Medicine Unit, Veneto Institute of Oncology IOV-IRCCS, 35128 Padua, Italy; 5 Nuclear Medicine Unit, Oncological Medical and Specialists Department, University Hospital of Ferrara, 44124 Ferrara, Italy; 6 Nuclear Medicine Unit, University Hospital “Paolo Giaccone”, Via del Vespro 129, 90127 Palermo, Italy; 7 Nuclear Medicine Department, ASP 1-P.O. San Giovanni di Dio, 92100 Agrigento, Italy; 8 Nuclear Medicine Department, Fondazione IRCCS Ca’Granda, Ospedale Maggiore Policlinico, via Francesco Sforza 35, 20122 Milan, Italy; 9 Department of Biomedicine and Prevention, University of Rome “Tor Vergata”, 00133 Rome, Italy; 10 Interdisciplinary Department of Medicine, University of Bari Aldo Moro, Piazza Giulio Cesare 11, 70124 Bari, Italy; 11 Nuclear Medicine Unit, S. Antonio Abate Hospital, 54027 Trapani, Italy; 12 Department of Medicine DIMED, University of Padua, 35128 Padua, Italy; 13 Nuclear Medicine Unit, Department of Biomedical and Dental Sciences and of Morpho-Functional Imaging, University of Messina, 98125 Messina, Italy; 14 Department of Experimental and Clinical Medicine, “Magna Graecia” University of Catanzaro, 88100 Catanzaro, Italy

**Keywords:** non-FDG PET criteria, PSMA-RADS, miTNM, Pro-PET, PRIMARY, PPP, RECIP 1.0, SSTR-RADS, NETPET, NAFCIST, FuMeGa

## Abstract

Background: in recent years, the role of positron emission tomography (PET) and PET/computed tomography (PET/CT) has emerged as a reliable diagnostic tool in a wide variety of pathological conditions. This review aims to collect and review PET criteria developed for interpretation and treatment response assessment in cases of non-[^18^F]fluorodeoxyglucose ([^18^F]FDG) imaging in oncology. Methods: A wide literature search of the PubMed/MEDLINE, Scopus and Google Scholar databases was made to find relevant published articles about non-[^18^F]FDG PET response criteria. Results: The comprehensive computer literature search revealed 183 articles. On reviewing the titles and abstracts, 149 articles were excluded because the reported data were not within the field of interest. Finally, 34 articles were selected and retrieved in full-text versions. Conclusions: available criteria are a promising tool for the interpretation of non-FDG PET scans, but also to assess the response to therapy and therefore to predict the prognosis. However, oriented clinical trials are needed to clearly evaluate their impact on patient management.

## 1. Introduction

Positron emission tomography (PET) and PET/computed tomography (PET/CT) have emerged in recent years as pivotal tools for the non-invasive assessment of a high number of pathological conditions. The most used tracer to perform this imaging modality is [^18^F]fluorodeoxyglucose ([^18^F]FDG) which can evaluate the glycolytic activity of cells [1,2], but other radiopharmaceuticals that explore different metabolic pathways or localize to particular targets because of specific binding interactions are available nowadays. The role of PET/CT imaging for the assessment of neoplasia has emerged in several steps of oncological patients’ work-up, such as in the diagnosis, staging, re-staging after therapy and follow-up. During the last 10 years, the use of many criteria has emerged in order to standardize the initial interpretation of oncological disease or their response after or during therapy. Imaging criteria can help in the interpretation of the scan, guiding the reader to better define its status or diagnosis. Moreover, since PET/CT is able to reflect different metabolic pathways of the tissues, functional changes can occur early in the course of therapy, preceding reduction in the size of tumors. Therefore, PET/CT can also be useful for the assessment of response to a specific therapy, thus guiding the future diagnostic and therapeutic work-up of the patient [3]. Different approaches measuring the response rate of neoplasms with morphological imaging modalities have been classically developed, such as the “Response Evaluation Criteria in Solid Tumors” (RECIST) [4]. Pointing our attention to [^18^F]FDG PET/CT, some specific response criteria have been formulated to improve its diagnostic predictive value, such as the EORTC, “PET/CT Criteria for early prediction of Response to Immune checkpoint inhibitor Therapy” (PECRIT), the “PET Response Evaluation Criteria for ImmunoTherapy” (PERCIMT) or the “PET Response Criteria in Solid Tumors” (PERCIST) [4,5,6,7]. In this scenario most of the PET criteria that have been proposed in the literature focus on [^18^F]FDG PET/CT, while only a small number of them were thought suitable for other radiopharmaceuticals. The aim of this systematic review is, therefore, to collect and discuss PET criteria developed for non-[^18^F]FDG probes in the oncological setting both for imaging interpretation and treatment response assessment.

## 2. Research Strategy

A bibliographic literature search up to 30 November 2022 was performed on three electronic databases (PubMed/MEDLINE, Scopus, Google Scholar) in order to find articles concerning the use of non-[^18^F]FDG PET criteria for the assessment of solid tumors. The search algorithms were different combinations of the following terms: “PET criteria”, “PET score”, “positron emission tomography”, “imaging interpretation”, “response”, “post-treatment evaluation criteria”, “osteosarcoma”, “Ewing sarcoma”, “bone tumor”, “brain”, “glioma”, “meningioma”, “breast cancer”, “pancreatic carcinoma”, “gastric cancer”, “esophageal cancer”, “anal cancer”, “GIST”, “cervical cancer”, “bladder cancer”, “head and neck squamous cell carcinoma”, “head and neck carcinoma”, “lung cancer”, “NSCLC”, “small cell lung cancer”, “mesothelioma”, “pleural mesothelioma”, “medullary thyroid cancer”, “thyroid cancer”, “anaplastic thyroid cancer”, “papillary thyroid cancer”, “adrenal gland tumor”, “neuroendocrine tumor”, “neuroendocrine”, “paraganglioma”, “prostate”, “prostate cancer”, “^18^F”, “^68^Ga”, “^11^C”, “MET”, “FET”, “DOPA”, “FCH”, “choline”, “DOTA”, “DOTATOC” “DOTATATE”, “DOTANOC”, “PSMA” and “NaF”. To identify supplementary eligible articles, the references of the retrieved articles were also screened for additional papers. Two reviewers (A.L. and J.G.) screened, retrieved, and selected data from each report. Original articles edited in the English language and performed on humans which evaluated non-[^18^F]FDG PET criteria for the evaluation of solid cancers were finally included in this review. Exclusion criteria were the assessment of [^18^F]FDG PET criteria and the assessment of semiquantitative parameters without the production of criteria. Review, meta-analysis, conference proceedings, case reports and case series were also excluded from the present analysis. The included studies were finally divided per cancer type classification, and they were also distinguished, if necessary, between interpretation and response assessment criteria in order to compare and evaluate mean differences between studies. From the included studies, the following information was extracted: characteristics of the studies (first author, year of publication), non-[^18^F]FDG PET criteria/score name, description of non-[^18^F]FDG PET criteria/score, type of cancer, radiopharmaceutical, sample size, and main findings.

## 3. Research Strategy Results

**Figure 1:**
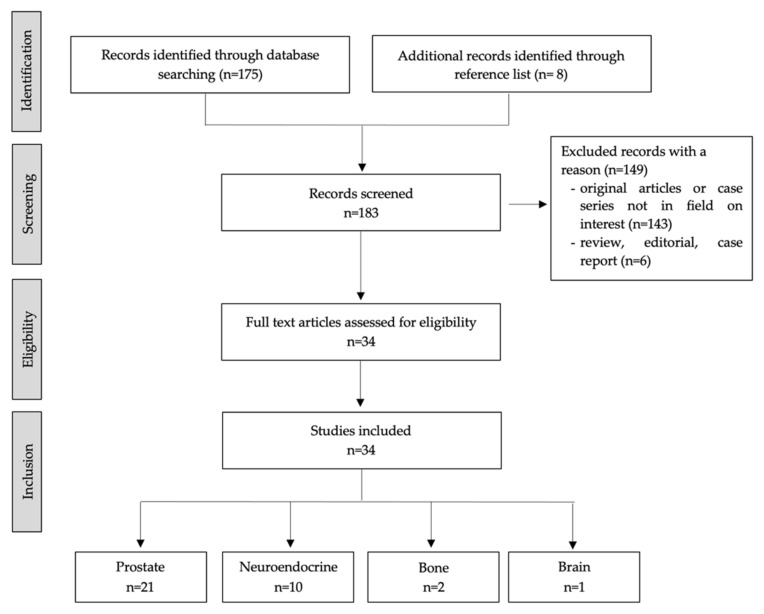
PRISMA flowchart of research strategy and studies selection.

The Preferred Reporting Items for Systematic reviews and Meta-analyses (PRISMA) flowchart of research strategy and studies selection is summarized in Figure 1. The literature search revealed 183 articles; among them, a total of 149 were excluded after reviewing titles, abstracts, and full texts because the reported data were not within the field of interest of this review. Therefore, 34 studies were considered suitable for the analysis and subsequently divided following different cancer types: Prostate Cancer (PCa) in 21 studies, Neuroendocrine tumors (NET) in 10, primary bone cancer in 2, and brain tumors in 1.

Finally, the research revealed 15 different non-[^18^F]FDG PET criteria: the European Association of Nuclear Medicine (EANM) criteria, the Prostate-Specific Membrane Antigen- Reporting and Data Systems (PSMA-RADS), the PROMISE (miTNM) criteria, the E-PSMA reporting system, the Pro-PET score, and the PRIMARY for PSMA PET imaging interpretation of PCa; PSMA PET Progression (PPP) and RECIP 1.0 as treatment response evaluation criteria for PCa. For NET, the PET-based Krenning score (KS), the somatostatin receptor (SSTR)-RADS and the NETPET grade were proposed for SSTR PET-based imaging interpretation and the selection of eligible patients for [^177^Lu]Lu-DOTA radioligand therapy (RLT), whereas MORE and ZP score were recently proposed for the assessment of response to RLT in NET patients. In primary bone cancer, the NAFCIST emerged as a response assessment criterion with [^18^F]sodium-fluoride ([^18^F]NaF) PET in patients affected by osteosarcoma or osteosarcoma-like tumor who underwent Radium^223^ dichloride (^223^RaCl_2_) therapy. Finally, the FuMeGa score was proposed to evaluate post-surgery response in high-grade glioma (HGG) patients with [^18^F]Fluorocholine.

**Figure 2:**
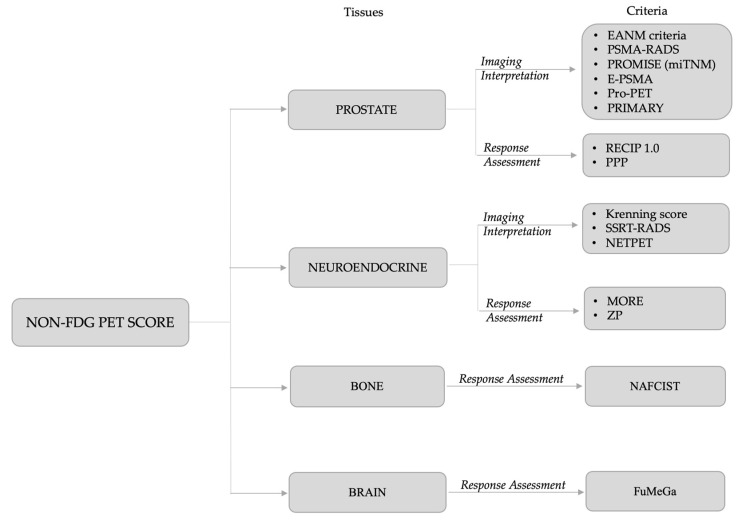
The fifteen non-[^18^F]FDG PET criteria retrieved from literature research and categorized following different tissues.

The proposed criteria per cancer type are reported in Figure 2 and detailed in Table 1. Data about the studies included in the review and their main findings are summarized in Table 2.

### 3.1. Prostate Cancer

#### 3.1.1. Interpretation Criteria: EANM Criteria, PSMA-RADS, PROMISE (miTNM), E-PSMA, Pro-PET and PRIMARY Score

On behalf of the EANM, Fanti et al. proposed the first standardized imaging interpretation system for [^68^Ga]Ga-PSMA PET. The criteria are structured as follows: first, “anomalous” findings, defined as suggestive radiotracer uptake above physiologic background, are recorded. Thus, all these sites are classified as “pathologic” for PCa, unless another explanation is possible. Third, the anatomic localization is considered (up to 5 lesions). However, in the same document, the authors observed a moderate interobserver agreement among multiple readers. Namely, inter-reader agreement for the presence of anomalous findings was 0.47 but became substantial when readers judged the anomalous findings as suggestive for a pathologic, uncertain, or non-pathologic image (K’s alpha: 0.64) [8].

The second proposed interpretation criterion, termed PSMA-RADS Version 1.0, was introduced by Rowe et al. in 2018 [9]. The PSMA-RADS can be applied on both individual target lesions (maximum of 5 per-scan) or on the overall impression of the imaging study. As shown in Table 3, PSMA-RADS is based on a 5-point scale that reflects the confidence of the interpreting imaging specialist that a given lesion represents a site of PCa, scoring from PSMA-RADS-1 (=definitively benign) to PSMA-RADS-5 (=high degree of certainty that PCa is present).

The interobserver reliability of PSMA-RADS was proved in a prospective study by Werner and colleagues, both on a per-lesion level and on an organ-based analysis by four readers with different levels of experience, evaluating 50 [^18^F]2-(3-{1-carboxy-5-[(6-18F-fluoro-pyridine-3-carbonyl)-amino]-pentyl}-ureido)-pentanedioic acid (DCFPyL) PET/CT scans. Notably, an excellent interobserver agreement was reported for lymph node assessment (ICC 0.79 95% CI, 0.66–0.89) and when considering the overall PSMA-RADS (ICC = 0.84 95% CI, 0.77–0.90) [22]. Similarly, in a retrospective single-center study of 56 PCa patients, patient- and lesion-based PSMA-RADS ratings showed great interrater reliability for bone metastasis (patient-based Cohen’s K = 0.88; lesion-based Cohen’s K = 0.82) [23]. More recently Letang et al. reported that PSMA-RADS had a significantly higher area under the curve (AUC) at receiver operating characteristic (ROC) analysis than the initial reading in clinical practice when assessing metastatic patients [24]. The PSMA-RADS-3 category (the most discussed in the literature) reflects the uncertainty level of the lesion to be compatible with PCa. In these regards, Yin et al. performed a longitudinal follow-up of 46 lesions considered indeterminate for PCa on [^18^F]DCFPyL PET/CT scans. During the follow-up, 58.7% of these lesions resulted true-positive for PCa. Moreover, the authors showed that PSMA-RADS-3A lesions were more likely to represent PCa than PSMA-RADS-3B ones [25]. Consistent with this evidence, another study reported that debatable lesions proved to have no clinical relevance in 84.6% of cases, and only 11% of equivocal PSMA-RADS-3B bone lesions were true positive [26]. In addition, Bhoil et al. reported that in the skeletal system only a minority of equivocal lesions findings will have characteristic changes in PCa involvement on follow-up imaging [27]. In the context of indeterminate lesions, an elevated prostate-specific antigen (PSA) value was shown in 81.9% of patients with true positive PSMA-RADS-3A lesions [28]. In this setting, the point-spread function (PSF) reconstruction, a modern reconstruction model with higher spatial resolution, was able to solve the diagnostic uncertainty of PSMA-RADS-3A lesions in 7.6% of cases, re-categorized as PSMA-RADS-4 [29]. PSMA-RADS categories were also correlated to maximum standardized uptake value (SUV_max_) by Mihatsch and colleagues, who reported a significantly higher SUV_max_ in PSMA-RADS-5 lesions. Furthermore, the challenging PSMA-RADS-3A lesions showed significantly lower SUV_max_ and SUV_peak_ compared to the PSMA-RADS-4 or -5 [30].

In 2019, Eiber et al. published the Prostate Cancer Molecular Imaging Standardized Evaluation, so-called PROMISE criteria. This molecular imaging TNM system (miTNM, version 1.0) consists of a standardized reporting framework for PSMA-ligand PET/CT or PET/magnetic resonance imaging (MRI) and provides a standardized system for the presence, location, and extent of local PCa and its pelvic and extrapelvic spread (Table 4) [10].

As suggested by the authors, the diagnosis of a PCa lesion should be assessed considering PSMA-uptake, location, and CT or MRI findings, as well as the specific clinical scenario. For this purpose, a standardized method for the PSMA expression level of tumor lesions was introduced (Table 5). Notably, the molecular imaging PSMA (miPSMA) score described the PSMA expression in relation to the mean uptake in the blood pool, liver (or spleen as reference organ instead of liver, for PSMA ligands with liver-dominant excretion e.g., [^18^F]PSMA-1007), and parotid gland. Scores 2 and 3 are considered typical for PCa lesions and favorable for PSMA-directed RLT [10].

In this setting, a prospective study determined the intra- and inter-observer agreement of [^68^Ga]Ga-PSMA-I&T PET/CT in 80 patients according to miTNM, reporting great interobserver agreement in the miN (Cohen’s K = 0.74) and miM (Cohen’s K = 0.84) categories. Differently, a lower interobserver agreement in the miT (Cohen’s K = 0.52) category was observed, especially in patients who had bladder or surrounding soft-tissue invasion [31]. Recently, Wang et al. evaluated the predictive role of preoperative miTNM from [^68^Ga]Ga-PSMA-11 PET in 187 patients with primary PCa who underwent radical prostatectomy. They showed that miTNM correlated with postoperative Gleason score, surgical margin status and time to biochemical recurrence [32]. Comparing [^68^Ga]Ga-PSMA-11 to [^18^F]PSMA-1007 PET in terms of miTNM staging, the miM staging was shown to be more concordant than the miT and miN; however, both tracers appeared widely exchangeable [33]. Koehler et al. evaluated the influence of a late scan of the pelvis at [^68^ Ga]Ga-PSMA-I&T PET/CT, reporting a change in 19.5% of the cases in miTNM in comparison with the early scan, and a change in staging in a not negligible number of subjects [34]. The inter- and intra-observer agreements were also evaluated according to the miTNM and PSMA-RADS in the study by Demirici et al. A substantial agreement was reported for miTNM system, while the PSMA-RADS showed almost-perfect agreement among readers. However, in the case of benign lesions authors observed more discordant results for PSMA-RADS than miTNM [35]. Finally, the 3 PSMA PET interpretation criteria (EANM, PSMA-RADS and PROMISE) demonstrated substantial to almost perfect interreader, intrareader, and intercriteria agreement in most situations as described by the group of Stanford [36].

In 2019, a consensus statement for standardized reporting of the PSMA-ligand PET was proposed by a panel of worldwide experts. The E-PSMA provides a structured report including what needs to be included in a report, considering different clinical settings, and including elements from the PROMISE and RADS systems [11].

More recently a dual-tracers scoring system combining [^68^Ga]Ga-PSMA and FDG was proposed for patients referred to [^177^Lu]Lu-PSMA RLT, termed Pro-PET score (Table 6). The scoring scheme, a 5-point categorical scale, is based on the single lesion that was the most FDG avid relative to its [^68^Ga]Ga-PSMA uptake (most discordant lesion). The concept proposal study retrospectively recruited 47 patients and showed that Pro-PET significantly correlated with the symptomatic response (*p* = 0.05), biochemical tumor marker response (*p* = 0.05), metabolic response (*p* = 0.001), anatomical response (*p* = 0.012), PFS (*p* = 0.03) and OS (*p* = 0.027). The trend observed showed unfavorable outcome when the disease shifted more towards the high grade of the Pro-PET scoring system [12].

In November 2022, a new score for primary PCa diagnosis based on intraprostatic PSMA-uptake pattern was published by Emmett and his group. The PRIMARY combined different pattern information and SUV_max_ assigned to each patient (Table 7). The specific patterns were described as follows: Pattern A, defined as diffuse transition zone (TZ) activity if centrally placed within the prostate, with no PSMA activity extending to the edge of the prostate margin; Pattern B, symmetrical central zone (CZ) activity with no PSMA activity extending to the prostate margin; Pattern C, focal TZ activity defined visually as more than twice background TZ activity; Pattern D, focal peripheral zone (PZ) activity. Table 7 shows the 5-point PRIMARY score classification [13].

The estimated AUC of the five-level PRIMARY score was 0.85 (95%CI: 0.81–0.89) and exceeded that of PI-RADS 0.76 (95%CI: 0.71–0.81) (*p* = 0.003). Furthermore, the authors showed a substantial inter-rater reproducibility for differentiating PRIMARY score low-risk from high-risk patterns between independent readers [13].

#### 3.1.2. Response Assessment: PPP and RECIP 1.0 Criteria

To date, two specific criteria for treatment response assessment in PCa patients have been developed. First, Fanti et al. proposed the PPP criteria [14] that defined progressive disease (PD) according to three different classes based on the appearance of new PSMA-positive lesions, clinical or laboratory data and, eventually, biopsy or imaging confirmation (Table 8).

Michalski et al. evaluated the feasibility of PPP criteria in patients undergoing [^177^Lu]Lu-PSMA therapy and their prognostic implications. The authors reported that inter-observer agreement was substantial, and that progression of disease evaluated by score was a significant prognostic marker for overall survival (OS) [37].

Considering the same scenario, Gafita et al. proposed a PSMA-PET response evaluation criteria for metastatic castration-resistant PCa (mCRPCa) patients treated with [^177^Lu]Lu-PSMA therapy, termed RECIP. Namely, the appearance of new lesions (=any new focal uptake of PSMA-ligand higher than surrounding background, and each tumor SUV_max_ > mean SUV_mean_) and changes in PSMA-VOL (the total positive PSMA volume) were combined to develop RECIP 1.0, which included the classifications of response to therapy presented in Table 9. The authors reported that RECIP-PD had a prognostic impact and the combination of PSA values and RECIP 1.0 criteria may result in a more reliable prognostic evaluation [15].

More recently, the same group compared the available response criteria for PCa, both specific (aPCWG3, PPP and RECIP 1.0) and non-specific (RECIST 1.1, aPERCIST), in the response evaluation of patients treated with [^177^Lu]Lu-PSMA. As a result, a better accuracy and inter-reader agreement were obtained with PSMA-specific criteria (PPP and RECIP 1.0). Moreover, a significant lower percentage of patients was defined as having PD according to RECIP 1.0 and these subjects had a higher risk of death in comparison with other response criteria [38].

### 3.2. Neuroendocrine Tumors

#### 3.2.1. Interpretation Criteria: PET-Based Krenning Score, SSTR-RADS, and NETPET

The most used method for determining SSTR-ligand uptake on imaging and the eligibility of NET patients for RLT is the Krenning Score (KS), based on the lesion with the highest uptake at [^111^In]Pentetreotide (OctreoScan) scintigraphy or SSTR-PET images (Table 10) [16]. However, the PET-based KS showed a higher sensitivity compared to [^111^In]Pentetreotide one, applied both to planar and single emission tomography (SPECT) scintigraphy [49,50,51].

Hope et al. performed a comparison of [^68^Ga]Ga-DOTA-TATE-based vs. [^111^In]Pentetreotide-based KS [39], reporting a detection rate of SSTR-positive disease (KS 2–4) of 23%, 38%, and 72% with [^111^In]Pentetreotide planar scintigraphy, SPECT and PET, respectively. Moreover, an influence of the size of the lesion on the KS for the three modalities and a correlation between SUV_max_ and KS were reported. These results imply that patients with lesions <2 cm would not have qualified for RLT based on [^111^In]Pentetreotide but appear as candidates on SSTR PET. The predictive role of KS from [^68^Ga]Ga-DOTA-NOC PET/CT was also demonstrated in lung carcinoids, with high sensitivity [40]. Finally, Menon and colleagues explored a dual-time-point [^68^Ga]Ga-DOTA-TATE PET/CT imaging protocol, but no significant changes in KS were reported [41]. The KS was created starting from [^111^In]Pentetreotide scintigraphy; therefore, reliable standards and criteria for SSTR PET are still lacking since many pitfalls can influence PET scans [52,53,54,55,56].

In this scenario, Werner et al. proposed a structured reporting system based on a 5-point scale for SSTR PET imaging, named SSTR-RADS Version 1.0, which might serve as a standardized assessment for both diagnosis and treatment planning in NET. The uptake levels for SSTR-RADS were established by a three-point qualitative assessment as shown in Table 11. For the clinical report, they suggested an overall interpretation of the SSTR PET scan with a minimum of clinical and imaging acquisition information and the number of lesions. Moreover, RLT may be considered in the case of an overall SSTR-RADS score of 4 or 5 (Table 12). The interobserver reliability of SSTR-RADS in [^68^Ga]Ga-DOTA-TOC PET/CT was evaluated by the same group, observing a high agreement in establishing the level of uptake and in the appropriateness of choosing RLT for both inexperienced and experienced readers [17].

In 2017, Chan et al. proposed a novel dual tracers SSTR/FDG PET grading scheme, the NETPET grade, a 5-point visual scale based on the characteristics of the reference lesions, grading from P1 (=purely SSTR-avid disease without FDG uptake in any lesions), to P5 (=significant FDG-positive/SSTR-negative disease) [18]. The score is presented in Table 13.

The authors found a statistically significant correlation between NETPET grade and OS at the univariate analysis. Furthermore, the score correlated with WHO 2010 histological grade (*p* < 0.00001) [18]. Some years later, the same group of authors validated the grading system in a bigger cohort of 319 metastatic/unresectable gastroenteropancreatic (GEP) NET patients, confirming its prognostic value in terms of OS and time-to-progression as well as with the histological grade [43]. The impact of NETPET was subsequently evaluated by other studies, and its potential use as a prognostic marker was confirmed [44,45] even in patients with bronchial neuroendocrine neoplasms (NENs) [46].

#### 3.2.2. Response Assessment: ZP and MORE

A standardized and reliable response assessment system after RLT is an unmet clinical need, as both morphological and functional imaging have shown limitations [57,58]. Recently, Zwirtz et al. compared the response evaluation with respect to OS in patients treated with at least two cycles of RLT introducing a new metabolic criterion, based on modified EORTC, so-called MORE criteria (Table 14). In addition, to identify other possible predictors of response, they generated two new combined parameters named ZP and ZPnormalized, using baseline CT and SSTR-PET data and summarized as follow [19]:

P (Target) = SUV_mean_ (Target) × Hounsfield Unit (HU) (Target)

ZPnormalized (Target) = normalized SUV_mean_ (Target) × HU (Target)

The concept proposal study, including 34 GEPNET patients, demonstrated that baseline ZP and ZPnormalized with overlapping sensitivity and specificity were the only predictive parameters of lesion progression after three RLT cycles. Moreover, patients who presented a progressive disease after the second cycle of RLT according to MORE criteria showed a significantly shorter OS [19,59].

**Table 14:** Response assessment criteria for NET patients underwent RLT.

		MORE	ZP
Category		Description	Description
Non PD	CR	Complete uptake disappearance in all lesions	No lesion (CT or PET)
	PR	≥25% reduction in the sum of SUV_max_ after more than one RLT cycle	≥25% reduction in the product of SUV_mean_ and HU
	SD	Does not meet other criteria	Does not meet other criteria
PD	PD	≥25% increase in the sum of SUV_max_ or at least one new lesion	≥25% increase in the product of SUV_mean_ and HU

Adapted from Zwirtz et al. [19]. Abbreviations: PD, progressive disease; CR, complete response; CT, computed tomography; PET, positron emission tomography; PR, partial response; SUV, standardized uptake value; RLT, radioligand therapy; HU, Hounsfield Unit; SD, stable disease.

### 3.3. Bone Primary Cancer: [^18^F]NaF PET Response Criteria in Solid Tumors (NAFCIST)

Kairemo et al. proposed an [^18^F]NaF PET imaging criterion for the response assessment of osteosarcoma in a Radium^223^ dichloride ([^223^Ra]Cl_2_) phase I clinical trial, termed “Na^18^F PET response Criteria in Solid Tumors” (NAFCIST) [20]. NAFCIST is based on the measurement of the mean SUV_peak_ in 1 cm^3^. The single most active bone avid lesion on each scan and the summed activity of up to the five most active ones (no more than two per organ) were considered for the category’s determination (Table 15). A significant correlation between the changes in NAFCIST and in alkaline phosphatase and bone alkaline phosphatase was shown, as well as a negative correlation between the cumulative dose of [^223^Ra]Cl_2_ and changes in NAFCIST, thus demonstrating that the more [^223^Ra]Cl_2_ administered, the more NAFCIST value decreased. Moreover, NAFCIST correlated with OS. Interestingly, in the same sample, PERCIST applied to [^18^F]FDG PET/CT did not show any significant correlations with outcomes [20]. In a subsequent work, the same authors applied radiomics to [^18^F]NaF PET imaging. A decrease in ^18^F^-^ concentration in metastatic areas was associated with an increase in intra-metastatic disorder after [^223^Ra]Cl_2_ treatment. Even with a small sample, these findings suggested that the more cycles of treatment were administered, the more radiotracer concentration and entropy decreased [47].

### 3.4. Glioma: Functional and Metabolic Glioma Analysis (FuMeGA)

García Vicente et al. presented the “Functional and Metabolic Glioma Analysis” (FuMeGA) score criteria for the visual interpretation of [^18^F]Fluorocholine PET/CT in patients with resected HGG, classifying complete versus incomplete metabolic tumor resection, as shown in Table 16 [21]. The prognostic value of the FuMeGa score on [^18^F]Fluorocholine PET/CT in HGGs was validated in a multicentric prospective study. Analyzing the postoperative score, significant differences were found for progression free survival (PFS) and OS for incomplete versus complete metabolic resections, respectively. The authors found that postoperative positive [^18^F]Fluorocholine PET/CT localizations correlated with the sites of tumor recurrence. Furthermore, on preoperative PET/CT, they observed that lesions with higher tracer uptake were followed by higher metabolic residual lesions after surgery [48].

## 4. Discussion

From its introduction in the 2000s, PET/CT gained space for the molecular imaging-based assessment of tumors in clinical practice [60,61]. In this scenario, many efforts have been made to develop standardized image analyses through the introduction of reliable, easy-to-use, and practical criteria. The existing scores were introduced mainly to assess treatment response, but the advent of new specific radiopharmaceuticals (e.g., PSMA-ligands compounds) underlined the need for a standardized method for imaging interpretation too. In this setting, PSMA showed to be the most promising tracer for PCa with high sensitivity and specificity, even if its role in different pathological conditions has been reported [62,63,64,65,66,67,68]. In this scenario, several criteria for interpreting PSMA-ligand PET were proposed. The first introduced EANM criteria demonstrated moderate consensus among readers, probably due to the absence of a scale, thus underlining the importance of categories in a structured reporting system. As consequence, the PSMA-RADS and the miTNM were proposed: the first, a 5-point visual scale, demonstrated a high interobserver agreement, even with different levels of experience; however, it lacks a reference uptake scale as introduced by the miTNM. The latter is the only score endorsed by the EANM guidelines and seems to pave the way for its introduction in large clinical trials [11]. However, considering the recent approvement of [^177^Lu]Lu-PSMA RLT for PSMA-positive mCRPCa patients [69], neither PSMA-RADS nor miTNM include treatment recommendations for this therapy needing warranting improvements. In this scenario, the recently proposed Pro-PET score combined both PSMA-ligand and [^18^F]FDG PET/CT performed before RLT to improve patients’ selection and serving as a prognostic marker. Among interpretation criteria, PRIMARY score is lastly emerged in literature as a promising criterion for the use of PSMA-ligand PET/CT also in the diagnosis of primary tumor. Considering instead the evaluation of response to treatment in PCa patients, it is usually performed by applying RECIST version 1.1 to CT, alongside the assessment of the PSA trend. However, these criteria have several limitations and are not enough to fully evaluate PCa patients [70,71]. [^18^F]Fluorocholine PET/CT or PSMA-ligands PET/CT are not currently recommended by most updated guidelines for this purpose, mainly due to the absence of prospective, randomized, large sample size trials [72,73,74,75]. In this scenario, Fanti et al. introduced the PPP score, based on PCWG3 guidelines principles, for the evaluation of progression in PCa patients. However, this criterion missed the definition of complete, partial, and stable metabolic patterns and does not consider changes in PSMA expression (as neuroendocrine differentiation) [76,77]. The traditional four response categories were re-introduced with RECIP 1.0 used to assess the response to [^177^Lu]Lu-PSMA. According to preliminary data, both criteria seem to correlate with OS and appear superior to assess responses compared to their non-specific counterpart (RECIST 1.1, aPERCIST).

Nowadays, theragnostic represents the driving force to introduce generalizable framework systems for standardized reporting as well as for treatment response assessment both for PCa and NET patients. In the last few years, the SSTR-RADS, structured in a reciprocal fashion of PSMA-RADS, were introduced to convey to the nuclear medicine scan reader the level of certainty that an equivocal finding is a site of disease, avoiding common pitfalls in interpreting SSTR imaging [52,53,54,55]. Such a framework also allowed identifying appropriate candidates for treatment with [^177^Lu]Lu-DOTA compounds. Our systematic review pointed out the few data available for the SSRT-RADS, probably due to the absence of dual-tracer incorporation into the score. Namely, dual PET imaging should be considered for all patients with a diagnosis of metastatic GEPNET (grade 2–3). In this regard, Chan and colleagues devised a novel scheme for dual SSTR/FDG grading, termed NETPET grade, which showed a significant correlation with OS, time to progression, and histological grade, serving as a predictor of outcome. Despite the introduction of RLT many years ago, no reliable treatment response assessment criteria were introduced [78,79]. In this setting, some authors evaluated different PET-derived parameters for response evaluation, with contradictory results [80,81]. The newly introduced scores that emerged in our review (MORE, ZP) need to be validated, but preliminary results demonstrated their possible role in prognostication and in prediction of response to RLT.

Other minor investigated non-[^18^F]FDG PET criteria deserve mention. The NAFCIST criteria, introduced to assess treatment response in patients undergoing [^223^Ra]Cl_2_ therapy on [^18^F]NaF PET imaging, seem to better correlated with outcome than PERCIST. Moreover, compared to RECIST, known to be suboptimal for the evaluation of osteosarcoma with calcified bone-forming tumors often not shrinking even if responding, NAFCIST could represent a more accurate method of categorizing osteosarcoma owing to its better ability in reflecting bone-forming component [20]. Finally, the FuMeGa criteria emerged as the first metabolic criteria introduced for post-operative PET interpretation in HGG patients. It is known that postoperative assessment in glioma patients is crucial in the imaging follow-up and for prognostic considerations [82]. Even for the evaluation of recurrence, PET imaging demonstrated its added value over MRI, given its ability to assess tumor metabolism and reduce pseudo-progression pitfalls [83]. However, [^18^F]Fluorocholine is not the standard of choice because amino acid tracers (such as [^11^C]MET, [^18^F]FET) demonstrated better diagnostic performance. However due to the limited availability of the amino acid PET tracers, [^18^F]Fluorocholine PET could be useful in blood–brain alterations, namely HGG.

To sum up, this systematic review showed a trend to standardization particularly evident in PCa because of its incidence, with promising evidence for SSTR-PET imaging and preliminary experiences for other oncological scenarios which warrant further and larger applications.

## 5. Conclusions

In conclusion, many criteria regarding the use of non-[^18^F]FDG PET imaging in oncology have been proposed in the literature, but the majority of them are not integrated into clinical practice. Even if more data are needed to clearly evaluate their impact on the management of patients, these criteria represent promising tools for the interpretation of PET scans and standardization of reporting, but also to assess the response to therapy and, therefore, to guide the prognosis.

## Figures and Tables

**Table 1:** The non-[^18^F]FDG PET criteria main characteristics and description.

Reference	Year	Radiotracer	Criteria	Description
Fanti et al. [8]	2017	PSMA-ligand	EANM criteria	All “anomalous” findings suggestive of recurrent PCa were noticed and categorized as “pathologic” unless another explanation could be hypothesized.
Rowe et al. [9]	2018	PSMA-ligand	PSMA-RADS	5-point scale framework reflecting the level of confidence in interpreting PSMA-PET imaging in PCa.
Eiber et al. [10]	2019	PSMA-ligand	PROMISE (miTNM)	Standardized reporting framework based on miTNM system for PSMA-ligand PET/CT or PET/MRI imaging in PCa.
Ceci et al. [11]	2021	PSMA-ligand	E-PSMA	The E-PSMA standardized reporting guidelinesprovide consensus statements to develop a structuredreport for PSMA-PET in PCa.
Adnan et al. [12]	2021	PSMA/FDG	Pro-PET	An integrated dual tracer PET/CT (PSMA and FDG) image scoring system for mCRPCa patients referred to [^177^Lu]Lu-PSMA therapy.
Emmett et al. [13]	2022	PSMA-ligand	PRIMARY	A 5-level score for the diagnosis of clinically significant PCa.
Fanti et al. [14]	2020	PSMA-ligand	PSMA PET Progression (PPP)	PSMA-driven response evaluation criteria in patients with metastatic PCa.
Grafita et al. [15]	2022	PSMA-ligand	RECIP 1.0	PSMA-driven response evaluation criteria in mCRPCa patients who underwent [^177^Lu]Lu-PSMA therapy.
Krenning et al. [16] *	1999	SSTR-ligand	Krenning	Visual interpretation criteria for evaluating NETs patients’ eligibility for [^177^Lu]Lu DOTA therapy.
Werner et al. [17]	2018	SSTR-ligand	SSTR-RADS	5-point scale framework reflecting the level of confidence of interpreting SSTR-PET imaging in NET assessing the eligibility to [^177^Lu]Lu-DOTA therapy.
Chan et al. [18]	2017	SSTR/FDG	NETPET	An integrated dual tracer PET/CT (SSTR and FDG) image scoring system for NET patients.
Zwirtz et al. [19]	2021	SSTR-ligand	MORE; ZP	Response evaluation criteria in NETs patients who underwent [^177^Lu]Lu-DOTA therapy.
Kairemo et al. [20]	2019	[^18^F]NaF	NAFCIST	Response evaluation criteria for high-risk osteosarcoma patients who underwent [^223^Ra]Cl_2_ therapy.
García Vicente et al. [21]	2020	[^18^F]Fluorocholine	FuMeGa	Visual interpretation criteria for post-surgery evaluation of HGG patients

* Adapted from [^111^In]Pentetreotide imaging to SSTR PET-imaging. Abbreviations: PSMA, prostate-specific membrane antigen; EANM, European Association of Nuclear Medicine; PCa, prostate cancer; RADS, Reporting and Data Systems; PET, positron emission tomography; mi, molecular imaging; CT, computed tomography; MRI, magnetic resonance imaging; FDG, fluorodeoxyglucose; mCRPCa, metastatic castration-resistant PCa; SSTR, somatostatin receptor; NET, neuroendocrine tumor; HGG, high-grade glioma.

**Table 2:** Selected studies main characteristics and key point by cancer type.

	Reference	Radiotracer(s)	n pts	Key Point
**Prostate**	Fanti et al. [8]	[^68^Ga]Ga-PSMA	104	EANM criteria showed a moderate interobserver agreement among multiple readers.
	Werner et al. [22]	[^18^F]DCFPyL	50	PSMA-RADS showed excellent interobserver agreement for lymph nodes and for the overall scan impression (ICC 0.79 and 0.84, respectively).
	Chiu et al. [23]	[^68^Ga]Ga-PSMA-11	56	PSMA-RADS ratings showed perfect interrater reliability to detect prostate bone metastasis. A SUV_max_ ratio (lesion-to-blood pool) > 2.2 presented a superior lesion detection rate and specificity when compared to PSMA-RADS.
	Letang et al. [24]	[^68^Ga]Ga-PSMA-11	53	PSMA-RADS had a significantly higher AUROC than the initial reading for the assessment of bone metastasis.
	Yin et al. [25]	[^18^F]DCFPyL	36	PSMA-RADS-3A lesions are more likely than PSMA-RADS-3B lesions to represent sites of PCa.
	Kuten et al. [26]	[^68^Ga]Ga-PSMA-11[^18^F]PSMA-1007	15	In intermediate and high-risk patients staged prior to radical prostatectomy, most PSMA-RADS-3B lesions are of no clinical relevance.
	Bhoil et al. [27]	[^18^F]PSMA-1007	203	A structured reporting with PSMA-RADS grading helps in the proper classification of lesions and standardization of reports.
	Garg et al. [28]	[^18^F]DCFPyL	28	A PSA level ≥ 0.6–1.0 ng/mL can be used as a marker for a high index of suspicion for true positivity in PSMA-RADS-3A lesions. A Gleason score ≥ 7 showed a higher rate of PSMA-RADS-3A lesion positivity.
	Khatri et al. [29]	[^18^F]DCFPyL	30	The PSF reconstructions allowed the re-categorization of a small number of indeterminate PSMA-RADS-3A soft tissue lesions as more definitive PSMA-RADS-4 lesions.
	Mihatsch et al. [30]	[^18^F]PSMA-1007	18	SUV_max_ was significantly higher in PSMA-RADS-5 lesions compared to all other categories. PSMA-RADS-3A lesions showed significantly lower SUV_max_ and SUV_peak_ compared to the entire PSMA-RADS-4 or -5 cohort.
	Gültekin et al. [31]	[^68^Ga]Ga-PSMA-I&T	80	The miN and miM categories showed almost perfect interobserver agreement (K = 0.93 and 0.94, respectively) as well as miM (K = 0.84).
	Wang et al. [32]	[^68^Ga]Ga-PSMA-11	187	Preoperative miTNM correlated with postoperative Gleason score, surgical margin status and time to biochemical recurrence.
	Hoberück et al. [33]	[^68^Ga]Ga-PSMA-11; [^18^F]PSMA-1007	46	In terms of miTNM staging, both [^68^Ga]Ga-PSMA-11 and [^18^F]PSMA-1007 appeared exchangeable, as no tracer outperformed the other.
	Koehler et al. [34]	[^68^Ga]Ga-PSMA-I&T	297	Additional late scans of the pelvis in [^68^Ga]Ga-PSMA-I&T PET/CT detected more lesions and an increasing contrast compared to early imaging, influencing the final miTNM-staging.
	Demirci et al. [35]	[^68^Ga]Ga-PSMA-11	133	The miTNM provides a high level of concordance among readers, with the highest agreement level in miM and lowest agreement in miT. PSMA-RADS showed almost-perfect agreement between readers in terms of scoring (ICC analysis). However, if the results were grouped as benign (score of 1/2), indeterminate (3), and malignant (4/5), Fleiss’ *κ* analysis showed a moderate level of agreement.
	Toriihara et al. [36]	[^68^Ga]Ga-PSMA-11	104	Comparing the 3 proposed interpretation criteria for PCa, intrareader agreement was moderate to almost perfect. The intercriteria agreement for each site was moderate to almost perfect.
	Adnan et al. [12]	[^68^Ga]Ga-PSMA-11 + [^18^F]FDG	47	The Pro-PET score significantly correlated with symptomatic, biochemical, metabolic, and anatomical responses, as well as with PFS (*p* = 0.03) and OS (*p* = 0.027).
	Emmett et al. [13]	[^68^Ga]Ga-PSMA-11	291	The estimated AUC of the PRIMARY score was 0.85 (95%CI: 0.81–0.89) and exceeded that of PI-RADS 0.76 (95%CI: 0.71–0.81) (*p* = 0.003). Sensitivity, specificity, PPV and NPV for PRIMARY score 3 to 5 (high risk patterns) vs. PRIMARY score 1,2 (low risk patterns) was 88%, 64%, 76% and 81%, compared to 83%, 53%, 69% and 72% for PI-RADS 3–5 vs. 2.
	Michalski et al. [37]	[^68^Ga]Ga-PSMA-11; [^18^F]PSMA-1007	46	Modified PPP criteria showed high reproducibility. A significant correlation was shown between progressive and non-progressive disease defined by PPP and OS.
	Gafita et al. [15]	PSMA-ligand	124	Patients with RECIP-PD had shorter OS compared to patients with RECIP-SD/PR
	Gafita et al. [38]	PSMA-ligand	124	PD patients according to RECIP 1.0 had a higher risk of death compared to non-PD, also in comparison with other response criteria (PPP, RECIST 1.1, aPERCIST)
**Neurendocrine**	Hope et al. [39]	[^68^Ga]Ga-DOTA-TATE	150	The detection rate of SSTR-positive disease (KS 2–4) resulted of 23%, 38%, and 72% with [^111^In]Pentetreotide planar scintigraphy, SPECT and PET, respectively. Lesion size did not affect SSTR PET-based KS.
	Purandare et al. [40]	[^68^Ga]Ga-DOTA-NOC	119	[^68^Ga]Ga-DOTA-NOC showed a high sensitivity for tumor detection (92.4%) and can help differentiate between typical and atypical carcinoid variants.
	Menon et al. [41]	[^68^Ga]Ga-DOTA-TATE	32	No significant change in KS was seen between the delayed scan compared to the standard one (1–1.5 h).
	Werner et al. [42]	[^68^Ga]Ga-DOTA-TOC	51	The interobserver agreement for overall scan impression based on SSTR-RADS was excellent (ICC, 0.88), as well as for lymph node and liver lesions (ICC, 0.91 and 0.77, respectively).
	Chan et al. [18]	[^68^Ga]Ga-DOTATATE + [^18^F]FDG	62	NETPET grade showed statistically significant correlation with OS at univariate analysis. NETPET grade was significantly associated with histological grade.
	Chan et al. [43]	[^68^Ga]Ga-DOTATATE + [^18^F]FDG	319	NETPET score significantly correlated with OS and TTP. NETPET showed both high inter-rater (kappa = 0.8) and intra-rater (kappa = 0.9) reliability.
	Hayes et al. [44]	[^68^Ga]Ga-DOTATATE + [^18^F]FDG	87	The dual-tracer PET classification was an independent predictor of OS (multivariate *p* = 0.016) and predicted PFS (univariate *p* = 0.030).
	Karfis et al. [45]	[^68^Ga]Ga-DOTATATE + [^18^F]FDG	85	Combined [^68^Ga]Ga-DOTATATE and [^18^F]FDG PET imaging classification reached high prognostic value in terms of PFS in GEPNET patients.
	Chan et al. [46]	[^68^Ga]Ga-DOTATATE + [^18^F]FDG	38	The NETPET score and histology were significantly correlated with OS (*p* = 0.003, *p* = 0.01)
	Zwirtz et al. [19]	[^68^Ga]Ga-DOTA-TATE/TOC	34	Only patients showing progressive disease after two PRRT cycles according to MORE criteria had a worse prognosis, while baseline ZP and ZPnormalized performed best in predicting lesion progression after three cycles of PRRT.
**Bone**	Kairemo et al. [20]	[^18^F]NaF	18	NAFCIST correlates with changes in bone alkaline phosphatase levels, cumulative dose of [^223^Ra]Cl_2_ and OS.
	Kairemo et al. [47]	[^18^F]NaF	18	Due to mixed response, neither PERCIST nor RECIST were able to predict response in osteosarcoma patients treated with [^223^Ra]Cl_2._ Radiomics can help in the assessment of intra-tumoral and inter-tumoral heterogeneity in the response to bone-forming osteosarcoma to [^223^Ra]Cl_2_ therapy.
**Brain**	García Vicente et al. [48]	[^18^F]Fluorocholine	47	Significant differences were found for PFS and OS between incomplete versus complete metabolic resections assessed by the FuMeGa score

Abbreviations: AUROC, area under the receiver operating characteristic curve; DCFPyL, 2-(3-{1-carboxy-5-[(6-18F-fluoro-pyridine-3-carbonyl)-amino]-pentyl}-ureido)-pentanedioic acid; GEP, gastroenteropancreatic; ICC, intraclass correlation coefficient; KS, Krenning score; mi, molecular imaging; NAFCIST, [^18^F]NaF PET response Criteria in Solid Tumors; NET, neuroendocrine tumor; NPV, negative predictive value; OS, overall survival; PCa, prostate cancer; PI, prostate imaging; PD, progressive disease; aPERCIST, adapted PET response criteria in solid tumors; PET/CT, positron emission tomography/computed tomography; PFS, progression free survival; PPP, PSMA PET progression; PPV, positive predictive value; PR, partial response; PRRT, peptide-receptor radionuclide therapy; PSA, prostate-specific antigen; PSF, point-spread function; PSMA, prostate-specific membrane antigen; RADS, Reporting and Data Systems; RECIST, Response Evaluation Criteria in Solid Tumors; SD, stable disease; SPECT, single photon emission tomography; SSTR, somatostatin receptor; SUV, standardized uptake value; TTP, time-to-progression.

**Table 3:** Summary of PSMA-RADS Version 1.0 for the interpretation of PSMA-PET imaging.

PSMA-RADS 1.0		
Category	Findings	Action
PSMA-RADS-1 (benign)		
PSMA-RADS-1A	Benign lesion characterized by biopsy or pathognomonic finding on anatomic imaging and without abnormal uptake	
PSMA-RADS-1B	Benign lesion characterized by biopsy or pathognomonic finding on anatomic imaging and with focal radiotracer uptake	
PSMA-RADS-2 (likely benign)	Equivocal uptake (focal, but low level such as blood pool) in soft-tissue or in a bone site atypical of PCa involvement	
PSMA-RADS-3 (equivocal)		Consider further work-up
PSMA-RADS-3A	Equivocal uptake in soft-tissue site typical of PCa involvement.	Lesion targetable: biopsy is suggested. Alternatively, follow-up imaging (either anatomic or PSMA-targeted PET/CT) after 3–6 months is recommended.
PSMA-RADS-3B	Equivocal uptake in bone lesion not definitive but also not atypical of PCa on anatomic imaging.	Comparison to other imaging modalities (bone scan, [^18^F]NaF PET, or tumor-protocol MRI images) or bone biopsy. Alternatively, follow-up imaging (either anatomic or PSMA-targeted PET/CT) after 3–6 months is recommended.
PSMA-RADS-3C	Lesions that would be atypical for PCa but have high levels of uptake. The likelihood of non-prostatic malignancy or another benign tumor is high.	Biopsy to histologically confirm the diagnosis is often preferred. Alternatively, organ-specific follow-up imaging may be done (e.g., liver-protocol MRI to evaluate possible primary hepatocellular carcinoma).
PSMA-RADS-3D	Lesion suggestive of malignancy on anatomic imaging but lacking uptake.	Consider non-prostatic malignancy, neuroendocrine PCa, and an uncommon case of prostate adenocarcinoma that fails to express PSMA. Biopsy to histologically confirm the diagnosis is often preferred; alternatively, organ-specific follow-up imaging may be done.
PSMA-RADS-4 (Pca highly likely)	Intense uptake in sites typical of PCa but lacking definitive findings on conventional imaging.	No biopsy confirmation will be needed, although tissue for genomic analysis (or other purposes) may be useful.
PSMA-RADS-5 (Pca almost certainly present)	Intense uptake in sites typical of PCand having corresponding findings on conventional imaging.	No biopsy confirmation will be needed, although tissue for genomic analysis (or other purposes) may be useful.

Adapted from Rowe SP et al. [9]. Abbreviations: PSMA, Prostate-specific membrane antigen; RADS, Reporting and Data Systems; PCa, prostate cancer; NaF, sodium fluoride; MRI, magnetic resonance imaging; PET/CT, positron emission tomography/computed tomography.

**Table 4:** miTNM version 1.0 system for Prostate Cancer Molecular Imaging Standardized Evaluation (PROMISE) using PSMA-PET tracer.

miTNM	
Class	Description
Local tumor (T)	
miT0	No local tumor
miT2	Organ confined tumor (unifocal or multifocal)
miT3	Non-organ confined tumor
miT3a	Extracapsular extension
miT3b	Tumor invading seminal vesicles
miT4	Tumor invading adjacent structures other than the seminal vesicles, such as external sphincter, rectum, bladder, elevator muscles, or pelvic wall
miTr	Presence of local recurrence after radical prostatectomy
Regional nodes (N)	
miN0	No positive regional lymph nodes
miN1a	Single lymph node region with lymph node metastases
miN1b	Multiple lymph node regions with lymph node metastases
Distant metastases (M)	
miM0	No distant metastasis
miM1	Distant metastasis
miM1a	Extrapelvic lymph nodes
miM1b	Bones
miM1c	Other sites

Adapted from Eiber M et al. [10]. Abbreviation: mi, molecular imaging.

**Table 5:** miPSMA score describing the PSMA-expression level of tumor lesions.

miPSMA Score			
Category	Score	PSMA Expression	Uptake
Negative	Score 0	No	Below blood pool
	Score 1	Low	Equal to or above blood pool and lower than liver (or spleen)
Positive	Score 2	Intermediate	Equal to or above liver (or spleen) and lower than parotid gland
	Score 3	High	Equal to or above parotid gland

Adapted from Eiber M et al. [10]. Abbreviation: mi, molecular imaging.

**Table 6:** The 5-point of Pro-PET score based on FDG avid relative to PSMA uptake of the reference lesion.

Pro-PET Score			
Score	PSMA-PET	FDG-PET	Description of Reference Lesion * (n° of Lesions)
Pro-PET 0	−	−	NA
Pro-PET 1	+	−	NA
Pro-PET 2a	+	+	FDG uptake less than PSMA (≤4 lesions)
Pro-PET 2b	+	+	FDG uptake less than PSMA (≤5 lesions)
Pro-PET 3a	+	+	FDG uptake equivalent to PSMA (≤4 lesions)
Pro-PET 3b	+	+	FDG uptake equivalent to PSMA (≥5 lesions)
Pro-PET 4a	+	+	FDG uptake more than PSMA (≤4 lesions)
Pro-PET 4b	+	+	FDG uptake more than PSMA (≥5 lesions)
Pro-PET 5	−	+	NA

* lesion most FDG avid relative to PSMA uptake. Adapted from Adnan et al. [12] Abbreviations: FDG, fluorodeoxyglucose; NA, not available; PSMA, prostate-specific membrane antigen.

**Table 7:** The schematic description of the PRIMARY score.

PRIMARY Score	
Score	Description
Score 1	No pattern, low grade activity.
Score 2	Diffuse TZ or symmetrical CZ activity without focal uptake (including diffuse TZ activity with irregular focal uptake not above background TZ activity).
Score 3	Focal TZ activity (must be visually above twice background TZ activity).
Score 4	Focal PZ activity
Score 5	Any pattern with SUV_max_ ≥ 12

Abbreviations: TZ, transition zone; CZ, central zone; PZ, peripheral zone; SUV, standardized uptake value.

**Table 8:** PSMA PET Progression (PPP) criteria for treatment response evaluation in patients with metastatic prostate cancer.

PPP Criteria		
Category	Progression Criterion	Description and Recommendations
A	≥2 new PSMA-positive lesions	≥2 new PSMA-positive distant lesions
B	1 new PSMA-positive lesion	1 new PSMA-positive lesion plus clinical or laboratory data. Recommended confirmation by biopsy or correlative imaging within 3 months from PSMA
C	No new lesions but size increase	Increase by ≥30% in size or uptake plus consistent clinical or laboratory data. Confirmation by biopsy or correlative imaging within 3 months of PSMA PET

Adapted from Fanti et al. [14]. Abbreviations: PPP, PSMA PET Progression; PSMA, prostate-specific membrane antigen; PET, positron emission tomography.

**Table 9:** Standardized framework for response evaluation criteria in PSMA PET/CT (RECIP) in mCRPCa patients.

RECIP 1.0	
Progression Criterion	Description
RECIP-CR	Absence of any PSMA-ligand uptake on PET after 12 weeks from RLT
RECIP-PR	Decline ≥ 30% in PSMA-VOL and no appearance of new lesions
RECIP-PD	Increase ≥ 20% in PSMA-VOL and the appearance of new lesions
RECIP-SD	Any condition but RECIP-PR or RECIP-PD

Adapted from Grafita et al. [15]. Abbreviations: CR, complete response; PR, partial response; PD, progression disease; SD, stable disease; PSMA, prostate-specific membrane antigen, PET, positron emission tomography; RLT, radioligand therapy; PSMA-VOL, total positive PSMA volume.

**Table 10:** PET-based Krenning score for eligibility of NETs patients to RLT.

PET-Based Krenning Score		
Category	Score	Interpretation
Negative	Score 0	no uptake
	Score 1	very low uptake
Positive	Score 2	uptake less than or equal to that of the liver
	Score 3	uptake greater than the liver
	Score 4	uptake greater than that of the spleen

Adapted from Krenning et al. [16].

**Table 11:** A three-point visual score for defining the uptake level of SSTR-avid lesion.

SSTR-Expression	
Level 1	≤bloodpool
Level 2	Uptake > bloodpool, but ≤physiological liver uptake
Level 3	uptake > physiological liver uptake

**Table 12:** Summary of SSTR-RADS version 1.0 system for the interpretation of SSTR-PET imaging and RLT eligibility.

SSTR-RADS				
Category	Findings	Uptake Level	Action	RLT
SSTR-RADS 1 (benign)	Benign lesion confirmed by biopsy or with a pathognomonic appearance on anatomic imaging			
SSTR-RADS 1A	Benign lesion, characterized by biopsy or by anatomic imaging and without any abnormal uptake	1		Not to be considered
SSTR-RADS 1B	Benign lesion, characterized by biopsy or by anatomic imaging but with increased (focal) uptake	2–3		Not to be considered
SSTR-RADS 2 (likely benign)	Soft-tissue site atypical of metastatic NET or equivocal uptake in bone lesion atypical for NET	1		Not to be considered
SSTR-RADS 3			Further work-up might be required	
SSTR-RADS 3A	Suggestive but not definitive for NET. Equivocal uptake in soft-tissue sites typical for NET metastases (e.g., regional lymph nodes).	1–2	Biopsy or initial fu imaging (SSTR-PET or whole-body MRI after 3 months), also depending on Ki-67/Grading.	Not to be considered
SSTR-RADS 3B	Suggestive but not definitive for NET. Uptake in bone lesions not atypical for NET.	1–2	Initial fu imaging (SSTR-PET or whole-body MRI after 3 months) might confirm diagnosis, also depending on Ki-67/Grading.	Single lesions: locoregional procedure. Increased number of lesions: RLT
SSTR-RADS 3C	Intense uptake in a site highly atypical for NET. Suggestive of an SSTR-expressing, non-NET benign tumor or malignant process.	3	Tissue confirmation of tumor histology should be considered.	Not to be considered
SSTR-RADS 3D	High likelihood of malignant NET lesion, but negative on an SSTR-PET scan (de-differentiated NET or another type of malignancy).	n/a	[^18^F]FDG PET should be considered. Tissue confirmation of tumor histology should be considered.	Not to be considered
SSTR-RADS 4 NET highly likely	Intense uptake in common site typical for NET lesion, but without confirmatory findings on anatomic imaging.	3	Further confirmation by biopsy might be not necessary.	To be considered
SSTR-RADS 5 NET almost certainly present	Intense uptake in site typical for NET with corresponding findings on conventional imaging.	3	Further confirmation by biopsy might be not necessary.	Definitely to be considered.

Adapted from Werner et al. [17] Abbreviations: SSTR, somatostatin receptors; RADS, Reporting and Data Systems; RLT, radioligand therapy; NET, neuroendocrine tumor; fu, follow-up; PET, positron emission tomography; MRI, magnetic resonance imaging; FDG, fluorodeoxyglucose.

**Table 13:** The 5-point of NETPET grade based on FDG avid relative to SSTR uptake of the reference lesion.

NETPET Grade				
Grade	SSTR-PET	FDG-PET	Description of Reference Lesion * (n° of Lesions)	Secondary Characteristics
P0	−	−	NA	
P1	+	−	NA	
P2a	+	+	FDG uptake less than SSRT (1–2 lesions)	
P2b	+	+	FDG uptake less than SSRT (≥3 lesions)	
P3a	+	+	FDG uptake equivalent to SSRT (1–2 lesions)	
P3b	+	+	FDG uptake equivalent to SSRT (≥3 lesions)	
P4a	+	+	FDG uptake more than SSRT (1–2 lesions)	
P4b	+	+	FDG uptake more than SSRT (≥3 lesions)	
	+	+	FDG+/SSRT- (1 lesion)	One additional lesion FDG > SSRT
P5	+	+	FDG+/SSRT- (1 lesion)	≥2 additional lesions FDG > >SSRT
	+	+	FDG+/SSRT- (≥2 lesions)	
	−	+	NA	

* lesion most FDG avid relative to SSRT uptake. Adapted from Chan et al. [18] Abbreviations: SSRT, somatostatin receptor; FDG, fluorodeoxyglucose; NA, not available.

**Table 15:** [^18^F]NaF response criteria in primary bone tumors (NAFCIST).

NAFCIST	
Category	Description
Complete metabolic response	Normalization of all lesions (target and non-target) to SUV less than the mean skeletal SUV and equal to the normal surrounding tissue SUV; verification with a follow-up study in 1 month if anatomical criteria indicate disease progression.
Partial metabolic response	>30% decrease in SUV_peak_ *; verification with follow-up study if anatomical criteria indicate disease progression.
Stable metabolic disease	Does not meet other criteria.
Progressive metabolic disease	>30% increase in SUV_peak_ *; >75% increase in total [^18^F]NaF burden of the five most active lesions; visible increase in the extent of [^18^F]NaF uptake; new lesions; verification with follow-up study if anatomical criteria indicate complete or partial response.

* Primary outcome determination is measured on the single most active lesion on each scan (not necessarily the same lesion). Secondary outcome determination is the summed activity of up to the five most intense lesions (no more than two lesions per organ). Adapted from Kairemo et al. [20]. Abbreviations: NAFCIST, Na^18^F PET response Criteria in Solid Tumors; Na^18^F, sodium fluoride-18; SUV, standardized uptake value.

**Table 16:** Functional and Metabolic Glioma Analysis (FuMeGa) criteria for post-operative assessment of HGG patients.

FuMeGa		
Category	Score	Description
Metabolic complete resection	Score 1	no uptake
	Score 2	faint uptake (lower than the contralateral skull) around the resection cavity
Incomplete metabolic resection	Score 3	moderate uptake around the resection cavity (like the contralateral skull)
	Score 4	moderate/high uptake around the resection cavity with focal deposits (unique)
	Score 5	moderate/high uptake around the resection cavity with focal deposits (multiple)

Adapted from García Vicente et al. [21].

## Data Availability

Not applicable.
